# Insights into Plastic Degradation Processes in Marine Environment by X-ray Photoelectron Spectroscopy Study

**DOI:** 10.3390/ijms25105060

**Published:** 2024-05-07

**Authors:** Tiziano Di Giulio, Giuseppe Egidio De Benedetto, Nicoletta Ditaranto, Cosimino Malitesta, Elisabetta Mazzotta

**Affiliations:** 1 Laboratorio di Chimica Analitica, Dipartimento di Scienze e Tecnologie Biologiche e Ambientali (Di.S.Te.B.A.), Università del Salento, Via Monteroni, 73100 Lecce, Italy; tiziano.digiulio@unisalento.it (T.D.G.); cosimino.malitesta@unisalento.it (C.M.); 2 Laboratorio di Spettrometria di Massa Analitica ed Isotopica, Dipartimento di Beni Culturali, Università del Salento, Via Monteroni, 73100 Lecce, Italy; giuseppe.debenedetto@unisalento.it; 3 Dipartimento di Chimica, Università degli Studi di Bari Aldo Moro, Via Orabona 4, 70126 Bari, Italy; nicoletta.ditaranto@uniba.it; 4 CSGI (Center for Colloid and Surface Science) Bari Unit, Università degli Studi di Bari Aldo Moro, Via Orabona 4, 70126 Bari, Italy

**Keywords:** X-ray photoelectron spectroscopy (XPS), plastic fragmentation, plastic degradation, low-density polyethylene (LDPE), polystyrene (PS), environment, marine environment

## Abstract

The present study employs X-ray photoelectron spectroscopy (XPS) to analyze plastic samples subjected to degradation processes with the aim to gain insight on the relevant chemical processes and disclose fragmentation mechanisms. Two model plastics, namely polystyrene (PS) and polyethylene (PE), are selected and analyzed before and after artificial UV radiation-triggered weathering, under simulated environmental hydrodynamic conditions, in fresh and marine water for different time intervals. The object of the study is to identify and quantify chemical groups possibly evidencing the occurrence of hydrolysis and oxidation reactions, which are the basis of degradation processes in the environment, determining macroplastic fragmentation. Artificially weathered plastic samples are analyzed also by Raman and FT-IR spectroscopy. Changes in surface chemistry with weathering are revealed by XPS, involving the increase in chemical moieties (hydroxyl, carbonyl, and carboxyl functionalities) which can be correlated with the degradation processes responsible for macroplastic fragmentation. On the other hand, the absence of significant modifications upon plastics weathering evidenced by Raman and FT-IR spectroscopy confirms the importance of investigating plastics surface, which represents the very first part of the materials exposed to degradation agents, thus revealing the power of XPS studies for this purpose. The XPS data on experimentally weathered particles are compared with ones obtained on microplastics collected from real marine environment for investigating the occurring degradation processes.

## 1. Introduction

Plastics are synthetic organic materials characterized by a high degree of chemical resistance, considerable plasticity, good tensile strength, and ease of processing [1,2,3]. However, such high versatility of plastics has led to their immoderate use [4] with their subsequent release into the natural environment due to mismanagement at the end of their lifecycle and their poor biodegradability [2]. It has been estimated that since the beginning of their mass production in the late 1940s, about 8 billion tons of plastic have been produced at the global scale, of which around 6 billion tons have been discarded [5].

**Figure 1:**
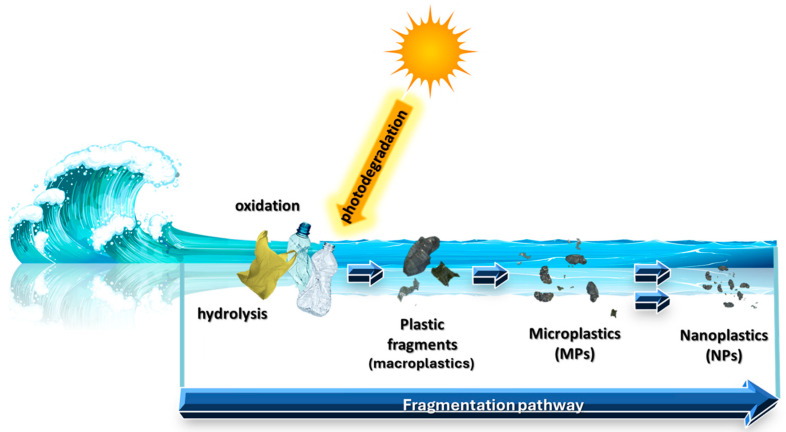
The effect of the fragmentation process: from everyday plastic items to dispersed micro- and nano-plastics.

The transition from macroplastics (MPs, >5 mm) to micro- (MiPs, <5 mm) and nano-plastics (NPs, ranging in size from 1 nm to 1 μm) [6] in the marine environment occurs due to physical erosion, which includes friction caused by wind, waves, and sand, as well as processes such as hydrolysis, oxidation, and photodegradation induced by solar radiation. All these processes contribute to the plastic fragmentation pathway, increasing the bioavailability of smaller particles (Figure 1). This is why plastics are pervasive in the oceans, ranging from coastal zones to offshore regions, including oceanic gyres and even remote areas like the Arctic [7].

In the environment, degradation processes facilitate plastic dispersion with synoptic-scale effects [8]. Indeed, it is known that these materials can negatively impact living organisms at many levels of biological organization, from individuals to populations and communities, through a wide range of mechanisms. There is a broad spectrum of effects on the individual organism, from the occurrence of inflammatory and phlogistic states to changes in gene expression and the onset of cancer disease. In marine environments, plastics can enter food webs through ingestion by animals, leading to bioaccumulation and/or biomagnification, or can also become carriers of toxic and hazardous substances (heavy metals or persistent organic pollutants), transported and spread through marine currents [5,9].

The timescale of plastic degradation in marine environments could be affected by different factors [10], and the dynamics of these processes are not completely clear, so gray areas remain [11]. It is recognized that hydrolysis and oxidation/photooxidation processes drive the fragmentation pathway of plastics [5,12] and different environmental agents can play a role [7]. Among them, air, water, and solar radiation are crucial [5,13,14]. Different mechanisms about the initiation step of plastic degradation have been hypothesized [15,16,17,18]. It has been proposed [15,16] that plastics can undergo oxidation in water when irradiated by UV radiation, due to the formation of charge transfer complexes (CTCs) as a result of the interaction between polymers and molecular oxygen (Equation (1)). Specifically, for polyolefins, the absorption of UV light could produce a triplet state able to interact with oxygen, producing a “proton loss” (H^+^) from the polymer, leading to radical species and hydrogen peroxide (a source of OH^.^ radicals) evolving in oxygen-containing functional groups [19,20].

(1) $$RH+O_{2}+hv={\;2R}^{.}$$

Another more recognized mechanism is related to the presence of oxygen-containing functional groups on the plastic surface, able to absorb UV light, leading new radical species. Among them, hydroperoxides can evolve in radical species, initiating plastic oxidation and then degradation (Equation (2)):

(2) $$ROOH+hv={\;RO}^{.}{+OH}^{.}$$

It is thus evident that the UV portion of solar radiation (100–400 nm), which represents only 5% of solar irradiance, is the one primarily involved in the oxidation process [4]. In nature, only the UV-A (315–400 nm) and UV-B (280–315 nm) portions succeed in reaching the earth’s surface, while the UV-C (100–280 nm) portion is filtrated by the atmosphere [19,20]. However, the resulting UV radiation could have, especially in the UV-B region, enough energy to produce the cleavage of the polymer chain [4]. In particular, the photooxidation mechanism [19] foresees a triggering step with the radiation absorption by chromophore groups present in the structure of the polymer [11,18,20] or resulting from contamination, defects, or additives during manufacturing [5,19]. If the energy of the radiation is sufficient, a cleavage of C-H could occur [4,13], with the formation of unstable radical species which react with molecular oxygen, producing peroxy-radicals and then hydroperoxy-radicals. These species can follow different reaction pathways, with the formation of oxygen-containing functional groups in the polymer that can act as chromophores, enabling a positive feedback mechanism [11], whereby the more these functional groups increase, the more prone plastics are to degradation [19].

Fragmentation processes also involve polymers that do not have chromophore groups and therefore should not undergo photooxidation processes under natural conditions, such as olefins (e.g., polypropylene and polyethylene). Although some plausible hypotheses have been provided [21,22], it is still not exactly clear how the degradation process kicks off and evolves [11].

X-ray photoelectron spectroscopy (XPS), alternatively known as electron spectroscopy for chemical analysis (ESCA), serves as a valuable tool for examining the surface chemistry of materials. It enables the determination of elemental composition, as well as the chemical and electronic states of atoms present at the surface of a material (within 10 nm thickness) [23,24,25]. It thus provides unique information about the material’s surface chemistry, which could be limiting in applications for which the bulk material chemical composition has to be investigated as the obtained information may not be representative, especially if the sample is heterogeneous or contains multilayers. However, given that the plastic surface directly interacts with the surrounding environment, insights from XPS analyses aid in comprehending the chemical processes involved in plastic degradation. The XPS technique has already been used to characterize the surface of polymeric/plastic materials [26,27,28,29,30] after weathering induced by plasma [31,32,33], discharge currents [34], mechanical stress/abrasion [35], high-energy radiation [36], hydrolysis in water [25], thermal treatment [37], biodeterioration [38,39,40], UV radiation [37,41,42,43], and natural environments [21,44]. Unfortunately, although the results obtained have been used to confirm and/or hypothesize degradation mechanisms, in most cases, they have not been validated through comparisons with control (untreated) or real-world samples. In addition, only a few studies that explore the synergistic effects of different factors on plastic degradation processes [35,37] are available.

In addition to XPS, Fourier-transform infrared spectroscopy (FTIR) and Raman spectroscopy are at times proposed for the chemical investigation of plastic degradation [45,46,47,48,49,50], although they may have limited spatial resolution, potentially missing localized changes [51].

Here, we use the XPS technique to provide insights into the degradation process of plastic materials, testing two model plastics, namely polystyrene (PS) and polyethylene (PE), analyzed before and after artificial UV radiation-triggered weathering, under simulated environmental hydrodynamic conditions, for different time intervals, with the aim to evidence chemical transformations related to degradation processes that possibly lead/contribute to fragmentation. The effect of UV radiation is discerned by analyzing samples treated under the same conditions but in the dark.

Importantly, plastics collected from real marine environments are analyzed by XPS for comparing results with ones collected on artificially weathered samples, thus possibly elucidating degradation processes occurring in real environments. Specifically, the selected plastics, LDPE and PS, belong to different groups. LDPE is the first commercially diffused polyolefin [52], frequently used in film applications and plastic container production [53]. PS is a thermoplastic material, the oldest artificial polymer discovered (1831) [54], widely used in automotive, electrical, and electronic connector systems [55]. Due to their wide use and subsequent improper disposal, LDPE and PS are extremely common in the environment, where they tend to remain and accumulate due to their high persistency [56]. The obtained results evidence that artificially aged plastics exhibit an increase in oxygen-containing functionalities, including hydroxyl, aldehydic/ketonic, and carboxylic groups. The plastic samples immersed in water without UV exposure show considerably fewer changes in surface composition, emphasizing the pivotal role of UV radiation in plastic degradation processes. The XPS analysis of plastics collected from real marine environments reveals the presence of similar functional groups thus allowing us to hypothesize degradation/fragmentation processes in real environments. Interestingly, only minor plastic modifications upon weathering treatments emerge from FT-IR and Raman spectroscopy investigations, further confirming the importance of investigating plastic surfaces in the study of fragmentation processes, it being the portion in contact with the surrounding environment and then with degradation agents.

## 2. Results and Discussions

### 2.1. XPS Analysis of Pristine and Artificially Weathered LDPE and PS

Both plastics, LDPE and PS, were artificially aged under simulated environmental conditions by exposure to UV radiation at a fixed temperature and relative humidity for different time intervals, as described in Section 3.2. Subsequently, pristine and as-treated plastic samples were analyzed by XPS, recording wide and high-resolution spectra.

An evident modification of plastics surface chemistry suddenly emerged from the comparison of the XPS wide spectra of pristine and weathered plastics (Figure S1), clearly revealing a gradual increase in the O 1s signal and the emergence of an N 1s signal upon the artificial weathering treatment on both LDPE (Figure S1A) and PS (Figure S1B). Oxygen and nitrogen content variation was quantified, respectively, by O/C and N/C atomic ratios, whose evolution with weathering time is illustrated in Figure 1.

**Figure 2:**
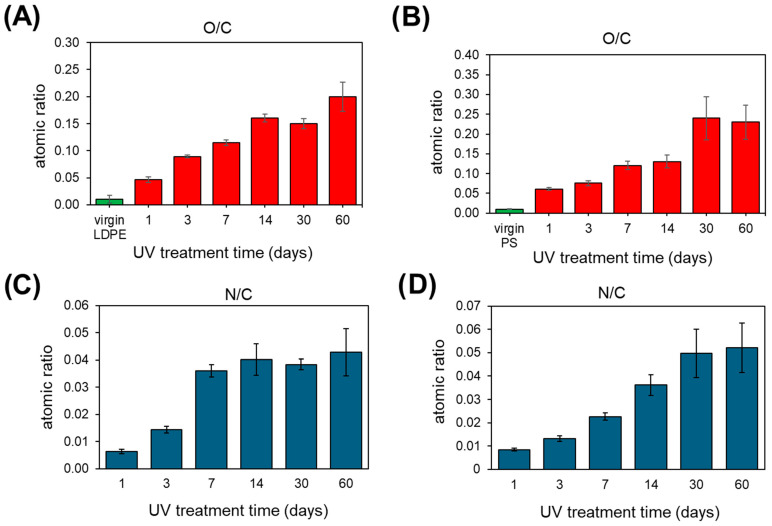
O/C and N/C atomic ratio calculated from XPS analysis of (**A**,**C**) LDPE and (**B**,**D**) PS samples exposed to artificial weathering process for different time intervals. For all the samples, three different measurement positions were analyzed (n = 3). Data are presented as mean (±s.d.).

Both LDPE and PS exhibited an increase in the O/C atomic ratio with an increasing treatment time up to 60 days (Figure 2A,B, respectively), suggesting the formation of oxygen-containing functional groups on the plastic surface, consistent with findings reported by other authors on UV-treated plastic materials [57]. The nitrogen content increase with the weathering was clearly demonstrated by the increase in the N/C atomic ratio in both LDPE and PS (Figure 2C,D, respectively) after the first 7–14 treatment days which then remained almost stable. The observed trend indicated that processes responsible for the increased nitrogen levels on the plastic surface likely occurred during the first weathering days. This finding may be attributed to gaseous nitrogen from dissolved air in water contaminating the sample surface over time, in agreement with what has been reported in similar studies on polypropylene [21] and low-density polyethylene [22].

**Figure 3:**
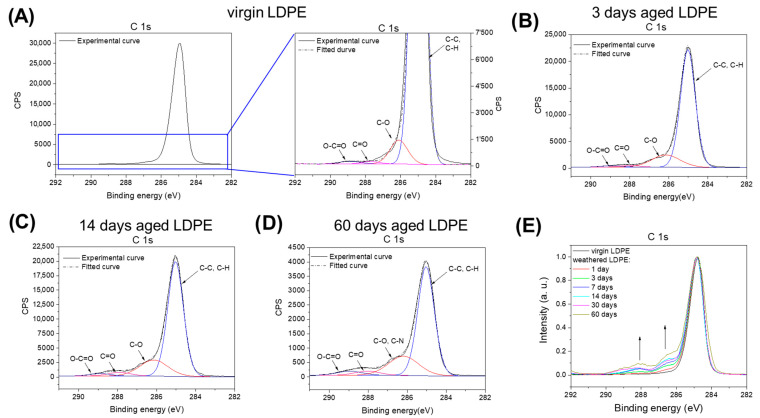
High-resolution spectra of C 1s signal recorded for: virgin LDPE (**A**), 3-, 14-, and 60-day-aged LDPE (**B**–**D**, respectively). (**E**) Comparison of C 1s signals recorded for LDPE at different weathering times.

**Scheme 1:**
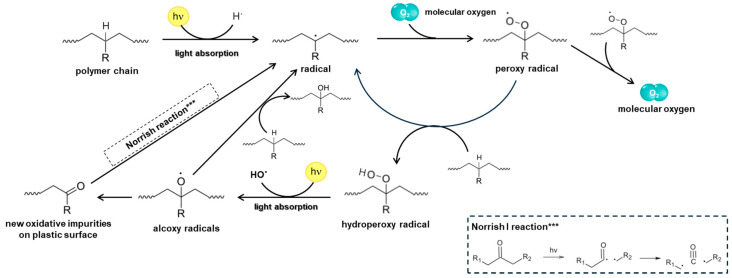
General photooxidation mechanism of PE (R H) and PS (R = Ph). Adapted from [19].

A more detailed examination of the high-resolution spectra offers valuable insights into the modifications of the treated plastics’ surface chemistry. Figure 3 presents the evolution of the C 1s signal for LDPE samples during the whole aging treatment. Although untreated LDPE plastics are not expected to show components associated with oxygenated carbon groups, minor oxidized components at higher binding energies can be detected (Figure 3A). Specifically, the fitting of the C 1s peak reveals one major component at 284.9 eV, attributed to hydrocarbon aliphatic species (C-C, C-H), alongside three minor components related to C-O, C = O, and O-C = O moieties at 286.2 eV, 287.8 eV, and 288.9 eV, respectively. This finding unexpectedly suggests their presence in virgin plastics of aldehydic/ketonic and carboxylic groups, which can act as chromophores absorbing UV radiation and triggering photooxidation, subsequently leading to polymer chain cleavage. Indeed, the presence of such functional groups is strongly related to processes involving the cleavage of chemical bonds within the polymer and thus to fragmentation. The absorption of UV radiation in the 290–320 nm range transfers sufficient energy to dissociate C-H bonds [19,43,58,59], thus forming free radicals that act as initiators of polymer degradation, as already hypothesized by several authors, following a multitude of chemical pathways [44,60,61]. Generally, hydroperoxides are initially generated as primary photoproducts. Once produced, they undergo decomposition via the rupture of the fragile O–O bond, yielding a macro-alkoxy and a hydroxyl radical. The alkoxy macroradical serves as a crucial intermediate in the process. This radical can undergo various pathways: β-scission resulting in the cleavage of the main chain to produce aldehydes, hydrogen abstraction without chain cleavage to generate hydroxyls, or a cage reaction between the pair of radicals formed, namely the macro-alkoxy radical and the hydroxyl radical. The latter reaction leads to the formation of chain ketones that undergo photochemical reactions by Norrish-type reactions [44,60,61,62,63] (Scheme 1).

Specifically, carbonyl groups are the primary light-absorbing species responsible for photochemical-induced degradation reactions in UV-exposed polymers. Such groups are not expected in polyolefins that are indeed considered inert due to the absence of moieties capable of absorbing sufficiently energetic radiation to induce bond cleavage [44,60,61,62]. Nonetheless, the identification of a small amount of such functional groups in native LDPE can be ascribed to impurities or defects, as reported by some authors [5,19], present in polyolefins, possibly resulting from their fabrication process and/or storage [64].

An analysis of the C 1s high-resolution spectra, recorded after different treatment times (Figure 3B–E), reveals that artificial aging results in an increase in components at higher binding energies, such as C-OH, C-N, C=O, and O-C=O, indicative of the amount of increase in oxidized chemical species. These results align with experimental evidence from other authors, who, using FTIR spectroscopy, indicated the formation of esters, lactones, aldehydes, ketones, and carboxylic acids on polyolefin structures after UV treatment [19]. Moreover, the observed increase in nitrogen content on the plastic surface suggests that reactive species (radicals) formed during photooxidation may react with nitrogen in the surrounding environment to produce new molecular species containing C-N bonds. Support for this hypothesis comes from the N 1s signal in the treated LDPE and PS samples centered around 400.5 eV (Figure S2), possibly attributed to amine groups or molecules containing the C-N moiety, although further investigation of this aspect is warranted.

Similar to what was observed for LDPE plastics, an increase in the oxidized carbon moieties is evident also from the analysis of the C 1s spectra of PS plastics at different treatment time intervals (Figure S3). Virgin plastics (Figure S3A) show a minor peak of the C-O component at 286.1 eV, in addition to the expected peaks at 284.9 eV and 291.5 eV, ascribed, respectively, to the aliphatic portion of the polymer and to π–π* shake-ups associated with the aromatic ring. The aging treatment leads to an increase in the C-O component and the appearance of new oxidized components (C-N, C=O, O-C=O) at a higher binding energy, resulting in a visible modification of the C 1s signal (Figure S3C, D, E). Specifically, after 14 days, C=O and O-C=O peaks appear at 287.6 and 288.7 eV, respectively, becoming more pronounced as the aging treatment progresses. From these results, it can be inferred that on virgin PS samples, the role of UV light-absorbing groups in initiating photodegradation is played by the aromatic ring [62,64]. Indeed, when subjected to UV irradiation, the benzene ring in polystyrene absorbs light photons, leading to the formation of polystyrene in the excited singlet state. Subsequently, the excited state transitions to the triplet state through intersystem crossing. Through intramolecular energy transfer, the excitation energy of the triplet state can be transferred to the C-H bond, leading to its breakage and the generation of radical species that initiate plastic degradation [62].

Nonetheless, the weathering treatment leads to the formation of additional functional groups that can contribute to radiation absorption determining photooxidation processes and possibly polymer chain rupture, as already hypothesized by other authors [47,65].

**Figure 4:**
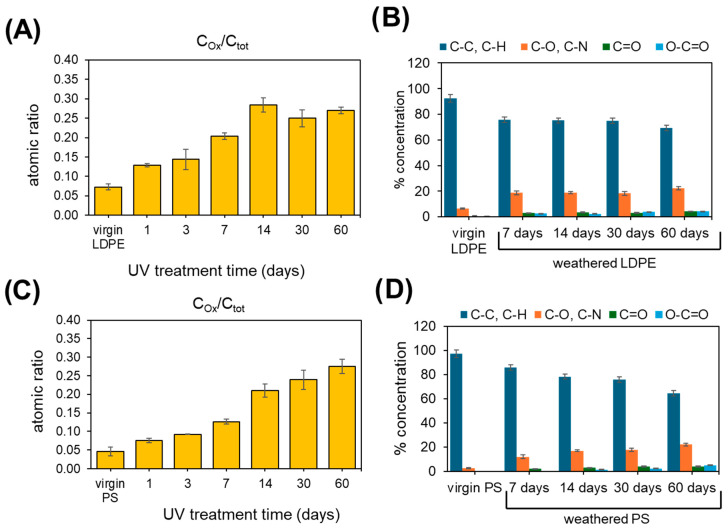
C_Ox_/C_tot_ atomic ratio obtained from fitted C 1s signal on (**A**) LDPE and (**C**) PS samples artificially weathered for different time intervals. C_Ox_ represents the area of the curve signal ascribed to oxidized components, and C_tot_ is the total area of C 1s signal. Concentration (%) of the components identified in the curve-fitted C 1s core-level spectra for (**B**) LDPE and (**D**) PS. For all the samples, three different measurement positions were analyzed (n = 3). Data are presented as mean (±s.d.).

From the comparison of the XPS results on LDPE and PS plastic samples, it emerges that, even initiated by different functional groups, UV-mediated oxidation processes lead to the formation of similar functional groups on both plastic surfaces. The increase in oxidized carbon species in both plastics is further evidenced by the progressive increase in the atomic ratio C_Ox_/C_tot_, where C_Ox_ represents the C 1s signal area ascribed to oxidized functionalities, and C_tot_ represents the total area of the curve-fitted C 1s core-level spectrum (Figure 4A,C). In LDPE samples, a certain stability is observed from 14 days of treatment onwards, contrarily to PS, in which the C_ox_/C_tot_ ratio continues to increase with the weathering treatment. To better investigate surface chemistry modifications, the percentage area of each component is quantified at different treatment times (Figure 4B,D). In LDPE samples (Figure 4B), C-O/C-N groups, which represent the main abundant oxidized component, readily reach stability already after 7 days of treatment, determining the overall steadiness of the C_ox_/C_tot_ ratio, although C=O and O-C=O slightly increase up to 60 days, passing, respectively, from 0.7 ± 0.1% and 0.6 ± 0.1% of the recorded signal on virgin LDPE plastics to 4.2 ± 0.4% and 4.1 ± 0.3% after a 60-day aging treatment. For PS (Figure 4D), in addition to the increase in the C-O component already present on virgin plastics, all the other functional groups formed with the weathering tend to increase up to the 60-day aging period, reaching 4.3 ± 0.3% and 5.2 ± 0.4%, respectively. This could be attributed to the lower air and water permeability of LDPE plastic compared to PS, as confirmed by contact angle measurements on virgin LDPE and PS plastics, indicating the greater hydrophobicity of polyolefins (Section 2.2).

For further assessing the role of UV radiation in oxidation/degradation processes and discerning it with respect to other possible contributions (especially of the water), weathering experiments on LDPE and PS are carried out under the same conditions in a dark environment. From the comparison of wide XPS spectra (Figure S4A,B), it is evident that plastics treated with UV show more pronounced modifications compared to those in the dark for the same time period. As demonstrated by the O/C atomic ratio, the surface of UV-treated plastics exhibits a significantly higher oxygen content, clearly demonstrating the role of UV radiation in promoting the formation of oxygenated species, likely because of photooxidation processes.

**Figure 5:**
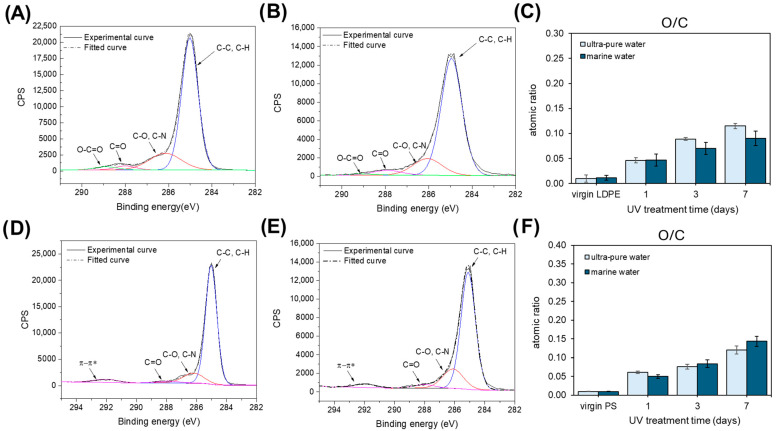
High-resolution spectra of C 1s signal recorded for (**A**) 7-day-aged LDPE in ultra-pure water; (**B**) 7-day-aged LDPE in seawater; (**C**) O/C atomic ratios recorded for LDPE samples treated under UV in ultra-pure water (light-blue column) and marine water (dark-blue column); (**D**) 7-day- aged PS in ultra-pure water; (**E**) 7-day-aged PS in seawater; (**F**) O/C atomic ratios recorded for PS samples treated under UV in ultra-pure water (light blue column) and marine water (dark blue column).

In marine environments, the effect of seawater composition is expected to influence plastic degradation processes, along with UV radiation. With the aim to ascertain this contribution, the weathering treatment is carried out under the same conditions but in seawater instead of fresh ultra-pure water and the collected results are compared (Figure 5). For both LDPE and PS samples, the similarity of C 1s signals recorded upon 7 days of treatment in fresh and marine water (Figure 5A,B,D–E, respectively) reveals that the aging process proceeds similarly under the two conditions, thus suggesting that, at least within this time interval, the seawater composition does not significantly affect the plastic degradation process. The same can be inferred also from the evaluation of O/C atomic ratios, reported in Figure 5C,F for LDPE and PS, respectively, for 1-, 3-, and 7-day treatments. As revealed from the wide spectra reported in Figure S5, an inevitable salts adsorption on plastics surface occurs in marine water [66], evidenced by Na 1s and Si 2p signals, which could partially attenuate UV light exposure, determining a slight slowing down of the degradation process.

### 2.2. Plastics Characterization by FTIR and Raman Spectroscopy

**Figure 6:**
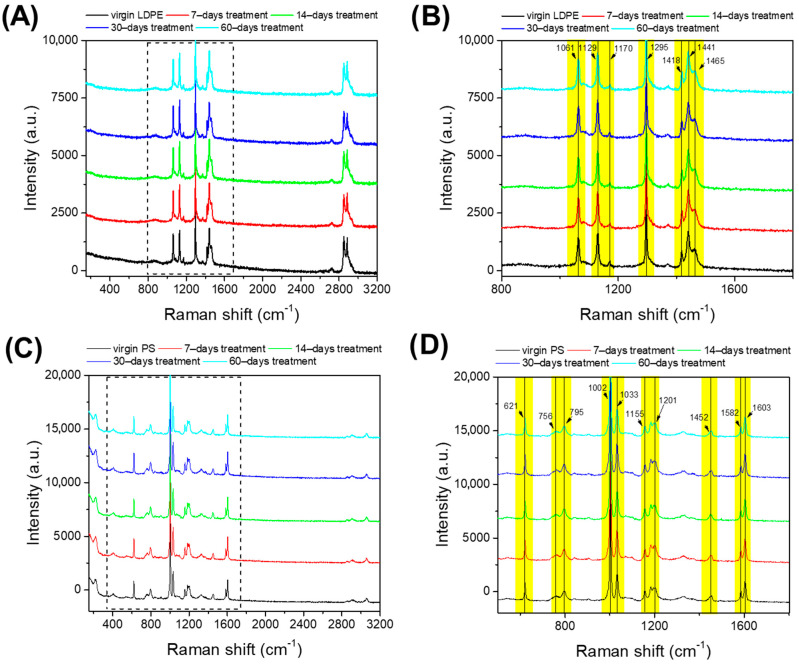
(**A**) Raman spectra of virgin (black line), 7-day-treated (red line), 14-day-treated (green line), 30-day-treated (blue line), and 60-day-treated (turquoise line) LDPE samples, recorded from 150 to 3200 cm^−1^; (**B**) detail of LDPE samples’ Raman spectra from 800 to 1800 cm^−1^. (**C**) Raman spectra of virgin (black line), 7-day-treated (red line), 14-day-treated (green line), 30-day-treated (blue line) and 60-day-treated (turquoise line) PS samples, recorded from 150 to 3200 cm^−1^; (**D**) detail of PS samples’ Raman spectra from 500 to 1800 cm^−1^.

The Raman spectra of LDPE samples (Figure 6A,B) align well with the existing literature [67,68], displaying characteristic bands centered at 1465, 1295, 1129, and 1061 cm^−1^ attributed to the all-trans–(CH2)_n_–structure. Additionally, bands at 2846 and 2887 cm^−1^ can be assigned to C–H (methyl) stretching vibrations (Figure 6A). The weak peak at ~2725 cm^−1^ suggests wavenumbers in the range 1400–1495 cm^−1^ (–CH_2_− bonds) [68]. The consistent presence of bands at 1465, 1295, 1129, and 1061 cm^−1^ indicates the material’s crystallinity, which remains unchanged even after UV treatment across all analyzed samples. Indeed, Raman spectroscopy analysis suggests the absence of alterations in the chemical properties of artificially aged LDPE samples.

Figure 6 (C and D) illustrates the Raman spectra of pristine and UV-treated PS samples at different time intervals. Spectral peaks of polystyrene are evident at 1002 cm^−1^, corresponding to aromatic breathing, peaks are evident at 1033 cm^−1^ assigned to C-H bending, and there is an evident band at 1603 cm^−1^, corresponding to C=C aromatic ring stretching [69]. A typical aromatic ring deformation occurs at 621 cm^−1^. Overlapping bands at 3061 cm^−1^ (Figure 6C) can be ascribed to C–H bonds stretching on the benzene ring. Consistently, no significant spectra variations ascribable to the weathering treatment are observed on the analyzed PS samples, as in the case of LDPE. This can be explained by the effective sampling depths of Raman spectroscopy, which range from 20 to more than 50 μm (depending on the numerical aperture of the lens and the laser wavelength), potentially limiting the ability to obtain local-level information (surface) [51].

Table 1 summarizes the band assignments for the Raman spectra of LDPE and PS samples made based on the literature [50,68,70,71,72].

**Figure 7:**
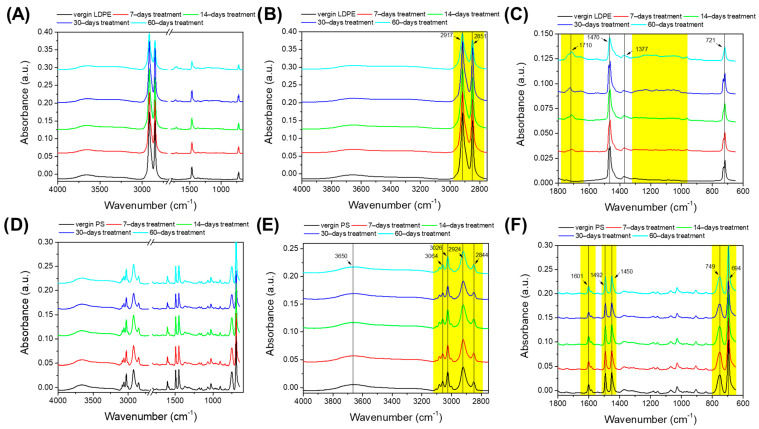
(**A**) FTIR spectra of virgin (black line), 7-day-treated (red line), 14-day-treated (green line), 30-day-treated (blue line), and 60-day-treated (turquoise line) LDPE samples, recorded from 400 to 4000 cm^−1^; (**B**) detail of LDPE samples’ FTIR spectra from 2750 to 4000 cm^−1^. (**C**) Detail of LDPE samples’ FTIR spectra from 650 to 1800 cm^−1^. (**D**) FTIR spectra of virgin (black line), 7-day-treated (red line), 14-day-treated (green line), 30-day-treated (blue line), and 60-day-treated (turquoise line) PS samples, recorded from 650 to 4000 cm^−1^; (**E**) detail of PS samples’ FTIR spectra from 2750 to 4000 cm^−1^; (**F**) detail of PS samples’ FTIR spectra from 2750 to 4000 cm^−1^.

The FTIR spectra of LDPE samples (Figure 7A–C) reveal typical bands at 1377 cm^−1^ (a distinctive band for polyolefins), 1470, and 2917 cm^−1^ attributed to -CH_3_ symmetric deformation vibrations, bending deformation, and -CH_2_ asymmetric stretching, respectively [73]. In the artificially weathered samples, a slight increase in the absorbance signal is noticeable in the range from 900 to 1300 cm^−1^, particularly in plastics treated for 30 and 60 days, consistent with observations made by other researchers [74]. Simultaneously, alterations in the region from 1550 to 1800 cm^−1^, accompanied by an increase in the intensity of the peak centered at 1710 cm^−1^ (associated with C=O stretching), are also observed. This indicates an increase in C=O functional groups in the plastic composition, in agreement with the XPS analysis results. Furthermore, with prolonged weathering treatment, a decrease in the intensity of the peaks at 2917 and 2851 cm^−1^ (related to C-H vibration) and at 1470 cm^−1^ (C-C bending) is noted. This corroborates findings from XPS analyses, which demonstrated a reduction in the aliphatic component of the polymer with increasing treatment durations.

Figure 7D–F illustrates the infrared absorption spectra of polystyrene samples showing several characteristic absorption peaks [75]. There are two evident peaks at 3064 and 3026 cm^−1^ related to the aromatic C-H stretching vibration and three main peaks at 1601, 1492, and 1450 cm^−1^ due to the aromatic C=C stretching vibration [75]. The absorption peaks at 749 and 694 cm^−1^ correspond to C-H out-of-plane bending vibration absorption. Minor peaks at 2924 and 2844 cm^−1^ could be related to the existence of methylene groups deriving from the synthesis procedure. In addition, the band at 3650 cm^−1^ is for the stretching vibration of O-H, which indicates the existence of hydroxyl, in agreement with what has been observed by the XPS analysis. No significant alterations were observed in the artificially weathered PS samples compared to the pristine samples, except for a slight decrease in peak intensities at 1492 and 1450 cm^−1^, likely associated with the loss or opening of aromatic rings, consistent with observations from XPS analyses.

The results from Raman and FTIR spectroscopic analyses further highlight the significance of XPS investigations in studying plastic degradation processes as the most pronounced changes occur in the surface portion of plastics.

### 2.3. Contact Angle Measurements on Artificially Weathered LDPE and PS Samples

Virgin and artificially weathered LDPE and PS samples were characterized through contact angle measurements. As reported in Figure S6, angles of 92.1° ± 0.9 and 81.4° ± 1.3 were recorded for LDPE and PS, respectively, in perfect agreement with the literature findings [31,76,77,78,79].

For both plastic samples, a gradual decrease in the measured contact angles was observed with an increasing aging time, with the resulting angles equal to 71.9° ± 5.3 and 73.2° ± 2.7 for LDPE and PS, respectively, after the 60-day treatment period. The contact angle values measured at all tested aging times are listed in Figure S6C.

The observed trend in the contact angles indicates a modification of the plastics’ surface composition towards increased hydrophilicity, in agreement with the XPS results. The increase in oxidized carbon functionalities (carboxylic, aldehydic, ketonic, and carboxylic groups) can be indeed correlated with greater hydrophilicity compared to the aliphatic portion of the polymer (C-C, C-H).

### 2.4. XPS Analysis of Naturally Weathered LDPE and PS Samples Collected from Real Marine Environment (“La Strea beach”, Porto Cesareo, Italy)

Samples of LDPE and PS, namely a bag and a sheet of LDPE and a spoon and a cup of PS were collected from a marine environment, as detailed in Section 3.2, and analyzed by the XPS technique. The resulting data were compared with those obtained from the artificially treated plastics.

**Figure 8:**
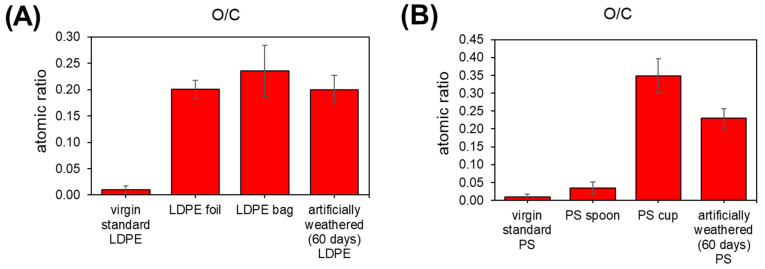
Comparison of O/C atomic ratios calculated for (**A**) LDPE and (**B**) PS samples collected from marine environment and artificially weathered for 60 days. For all the samples, three different measurement positions were analyzed (n = 3). Data are presented as mean (±s.d.).

The O/C atomic ratio was estimated for all samples and compared with values calculated for the 60-day artificially weathered LDPE and PS (Figure 8). For LDPE samples, O/C ratios are similar to the values obtained for plastics artificially weathered for 60 days. This finding, although it cannot exhaustively elucidate the degradation timescale of real samples, can give an indication of persistence time in the environment, during which the plastic samples are exposed to the action of degradation/fragmentation agents [5,11,80]. In the case of PS, different results emerge for the spoon and cup samples, with a remarkably higher oxygen content on the surface of the cup. Indeed, the possible longer aging of the cup material is evident to the naked eye by its yellowing. Specifically, the O/C ratio for the PS cup is higher than that evaluated for the PS subjected to 60 days of UV treatment, possibly suggesting a prolonged exposure of the plastic to the marine environment.

**Figure 9:**
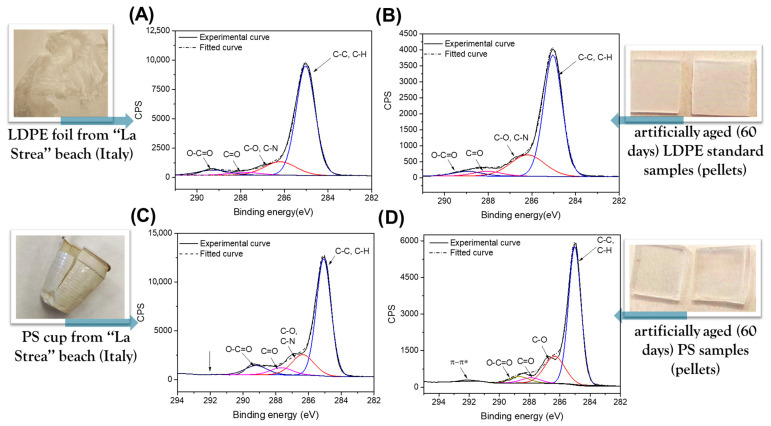
High-resolution spectra of C1s signal recorded for LDPE foil collected from real environment (**A**), 60-day artificially weathered LDPE sample (**B**), PS cup collected from real environment (**C**), and (**D**) 60-day artificially weathered PS sample.

On the plastic samples collected from the environment, in addition to wide spectra (Figure S7), the high-resolution spectra of the C 1s region were recorded (Figure 9), comparing the results with those from the 60-day artificially aged standard samples. It is readily evident that similar functionalities can be detected on natural and artificial samples for both LDPE and PS, suggesting that similar reaction pathways characterize the artificial and the natural aging process for plastics. Of particular interest is the C 1s spectrum recorded for the PS cup. Indeed, although the same oxidized C1s components can be found in artificially aged PS standards, the absence of the shake-up contribution can be seen. This result further demonstrates the high oxidation/degradation degree of the cup. In fact, the aging process may be so advanced so as to cause the decrease in π–π* due to the detachment of small molecules containing phenyl rings from the PS backbone [41] or even to the opening of aromatic rings in the polymer chain, leading to the formation of mucodialdehyde groups, as previously reported by other authors [58]. In general, these changes are associated with the discoloration and yellowing of PS, as was observed on the real sampled cup.

## 3. Materials and Methods

Standard plastics sheets (20 × 30 cm) of different types (low-density polyethylene, LDPE, and polystyrene, PS) with a thickness of 1 mm were provided by Goodfellow^®^ (Pittsburgh, PA, USA). For the weathering experiments, plastic pellets of a size of around 1 cm^2^ were obtained from the sheets. Before their use, the protective films initially present on their surface were removed from the plastics. Plastic pellets were stored in plain paper containers when they were not used. Ultra-pure water with an ionic conductivity of 0.055 μS cm^2^ was used for the experiments.

### 3.1. Artificial Weathering Treatment

**Scheme 2:**
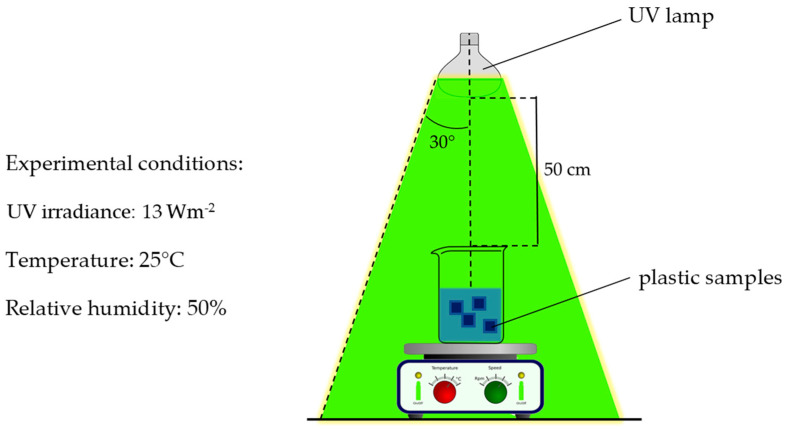
Schematic of the weathering treatment of the plastic samples; plastic pellets were immersed in ultra-pure water under agitation (400 rpm) and then were exposed to UV radiation for different periods.

A climatic chamber (Angelantoni, CHALLENGE 250 ^®^, Massa Martana, Perugia, Italy), equipped with a UV lamp (OSRAM Ultra-Vitalux^®^, Munich, Germany, 300 W 230 V E27), was used for the weathering treatment. The UV lamp provides a radiative power of 13.6 W and 3 W for UV-A and UV-B, respectively, selected for simulating solar radiation [57,81]. Plastic pellets were immersed in ultra-pure water (1%, *w*/*v*), and were irradiated by the UV lamp for different time intervals (Scheme 2). Alternatively, plastic pellets were immersed in marine water to assess matrices effects. In the climatic chamber, the samples were placed in a position where the light intensity in the UV range (λ = 280–400 nm) was 13 Wm^−2^ (10.95 Wm−2 and 2.15 Wm−2 for UV-A and UV-B, respectively), simulating the solar irradiance at Mediterranean latitudes [82,83]. The irradiation intensity was measured using a radiometer (PMA2100/light meter/photometer/UV meter, Solar Light Co., Glenside, PA 19038, USA). The climatic chamber allowed the continuous monitoring of the temperature and relative humidity, set respectively at 25 °C and 50% so that they were close to average Mediterranean environmental conditions, for the entire duration of the treatment. During treatment, the samples underwent stirring (400 rpm), and the water was periodically replaced after 24 h with fresh ultra-pure water. After treatment, the pellets were stored in paper containers in the dark until their analysis. Control experiments were carried out under the same conditions but in a dark environment.

### 3.2. Sampling of Naturally Weathered Plastics from Marine Environments

Naturally aged plastic samples were collected during March 2023 from La Strea Bay in Porto Cesareo (Italy) (40°16′ N 17°54′ E), a marine protected area on the Ionian coast. Plastic samples were washed with ultrapure water, identified through their labeling/code, and stored in plain paper containers until XPS analysis.

### 3.3. XPS Analysis of Plastic Samples

XPS measurements were conducted utilizing an AXIS ULTRA DLD (Kratos Analytical, Manchester, UK) photoelectron spectrometer, employing a monochromatic Al Kα source (1486.6 eV) operating at 150 W (10 kV, 15 mA). The analysis chamber maintained a base pressure of 5 × 10^−9^ torr. Survey scan spectra were obtained using a pass energy of 160 eV and a 1 eV step, while high-resolution spectra were acquired with a pass energy of 20 eV and a 0.1 eV step. The analysis area for each measurement was approximately 700 μm × 300 μm. A charge neutralization system was employed during data acquisition. Spectral processing was conducted using CasaXPS software (Casa Software Ltd.^®^, Teignmouth, USA, version 2.3.16), with the binding energy (BE) scale referenced to the Au 4f7/2 peak at 84.0 eV. The fitting of high-resolution spectra involved the application of the Shirley background and GL(30) line shape (a blend of Gaussian 70% and Lorentzian 30%) for all peaks. For quantitative analysis, the relative sensitivity factors (RSFs) from the CasaXPS^®^ library for the areas of the signals/regions were used. The following regions were recorded: C 1s, O 1s, and N 1s. Virgin plastics were analyzed for comparison with artificially and naturally aged plastic samples. The elemental content of the samples was expressed as atomic ratios such as O/C and N/C, where O, C, and N represent the area of O 1s, C 1s, and N 1s regions, respectively. Alternatively, the C_Ox_/C_tot_ atomic ratio was used to assess the changes in plastic composition due to the weathering, where C_Ox_ represents the area of the curve signal ascribed to oxidized components and C_tot_ is the total area of the C 1s signal. Surface charging was corrected considering adventitious C 1s (binding energy (BE) = 284.9 eV).

### 3.4. Plastics Characterization by Contact Angle Measurements

Contact angle measurements were performed with an open-source contact angle analyzer, using a smartphone [84]. The experimental apparatus consisted of a chamber containing a holder sample (a plate) located between a light source and a camera. The plastic sample was placed on the plate and a water droplet (2 μL) was dropped on the sample surface with a syringe. A high-quality image of drop was collected by the smartphone camera, and the contact angle was measured using ImageJ software (version 1.54d, Wayne Rasband, National Institutes of Health, Stapleton, MA, USA). For each sample, several pictures (n = 5) were recorded on different positions (n = 3) [85].

### 3.5. Plastics Characterization by Raman Spectroscopy

Raman analyses were performed using a Renishaw^®^ inVia apparatus (Renishaw GmbH, Pliezhausen, Germany) equipped with a Leica^®^ microscope (Leica Microsystems GmbH, Wetzlar, Germany) with 50×/20×/5× objectives and a 785 nm diode laser. System calibration was performed on the 520 cm^−1^ peak of an n-doped silicon wafer (laser power of 5%, acquisition time of 10 s and 4 accumulations).

The infrared spectra were obtained using a Cary 600 Agilent Technologies FTIR spectrometer (Agilent Technologies, Milano, Italy). The measurements were performed in attenuated total reflectance, and ATR-FTIR spectra were collected in the spectral region between 650 and 4000 cm^−1^, with a resolution of 4 cm^−1^ and averaging 32 scans. All spectra were ATR and baseline corrected.

## 4. Conclusions

In the present work, the XPS study of plastic materials is presented with the aim to gain insight into the mechanism responsible for plastic degradation/fragmentation, focusing on UV artificially weathered samples under simulated environmental conditions and samples collected in a marine environment.

The XPS analysis reveals an unexpected oxygen content already on virgin LDPE plastics possibly related to the presence of oxygen-containing functional groups due to impurities/defects that act as UV light-absorbing agents able to trigger photooxidation. This may explain why some plastics that, in principle, do not contain photoabsorbent groups undergo oxidation/hydrolysis processes that then lead to their fragmentation. The increase in oxidized components directly correlates with the aging of plastics. Essentially, the higher the presence of oxidized components, the more advanced the aging process. LDPE thus reveals to be more susceptible to surface oxidation than might be expected. This probably explains why polyethylene is one of most common microplastics found in estuaries and the marine environment.

Similarly, for the PS samples, the aging treatment results in an increase in the C-O component and the emergence of new oxidized components (C-N, C=O, O-C=O) at higher binding energies, thereby visibly altering the C 1s signal. Then, the weathering treatment induces the formation of additional functional groups capable of absorbing in addition to the native aromatic ring, rendering the plastic more susceptible to photooxidation and then to polymer chain rupture.

The analysis of the LDPE and PS samples treated in a dark environment demonstrates that oxidation occurs significantly faster in the presence of UV radiation. However, the contact with water under hydrodynamic conditions also induces detectable changes in the surface chemistry of plastics, leading to an increase in oxygen-containing functionalities.

The comparison of the XPS results for the LPDE and PS samples collected in a real marine environment reveals the same functionalities identified on the artificially aged samples for both LDPE and PS, suggesting that similar reaction pathways characterize the artificial and the natural aging processes for plastics.

The insights obtained from the XPS analysis are further highlighted from the comparison with FTIR and Raman investigations. While Raman and FTIR spectra indicate only slight changes in the plastic samples aged over different periods, XPS analysis provides a distinctive ability to reveal the chemical modifications of plastics’ surface, which represents a highly informative region of the samples directly interacting with the surrounding environment and degradation agents.

## Figures and Tables

**Table 1:** Raman mapping: wavenumbers (cm^−1^) and assignments of the Raman bands of LDPE and PS materials.

Raman Shift (cm^−1^)	Assignments (LDPE)	Assignments (PS)
3061		C-H aromatic ring stretching
2885	C-H (methyl) stretching	
2846	C-H (methyl) stretching	
1603		C=C aromatic ring stretching
1582		C=C aromatic ring stretching
1465	CH_2_ bending	
1441	CH_2_ bending	
1418	CH_2_ bending	
1295	CH_2_ twisting	
1201		Aromatic ring vibration
1170	CH_2_ rocking	
1155		C-C stretching
1129	C-C symmetric stretching	
1061	C-C antisymmetric stretching	
1033		C-H bending
1001		Aromatic breathing
798		C-H bending
621		Ring deformation

## Data Availability

Data is contained within the article or Supplementary material.

## Supplementary Materials

The following supporting information can be downloaded at: https://www.mdpi.com/article/10.3390/ijms25105060/s1.
