# Supplementary material for: Insights into Plastic Degradation Processes in Marine Environment by X-ray Photoelectron Spectroscopy Study
Source: Int J Mol Sci. 2024 May 7;25(10):5060. doi: 10.3390/ijms25105060 (PMC11121657; doi:10.3390/ijms25105060)
Supplement: Supplementary file 1 [file ijms-25-05060-s001.zip › ijms-2918691-supplementary.pdf]

# Insights into plastic degradation processes in marine environment by X-ray photoelectron spectroscopy study

**Tiziano Di Giulio[1], Giuseppe E. De Benedetto[2], Nicoletta Ditaranto [3,] Cosimino Malitesta[1], Elisabetta Mazzotta[1,*]**

[1] Laboratorio di Chimica Analitica, Dipartimento di Scienze e Tecnologie Biologiche e Ambientali (Di.S.Te.B.A.), Università del Salento, via Monteroni 73100 Lecce – Italy

[2] Laboratorio di Spettrometria di Massa Analitica e Isotopica, Dipartimento di Beni Culturali, Università del Salento, via Monteroni 73100 Lecce – Italy

[3] Dipartimento di Chimica and CSGI—Bari Unit, Università degli Studi di Bari Aldo Moro, Via E. Orabona 4, 70126 Bari, Italy

* Correspondence: elisabetta.mazzotta@unisalento.it

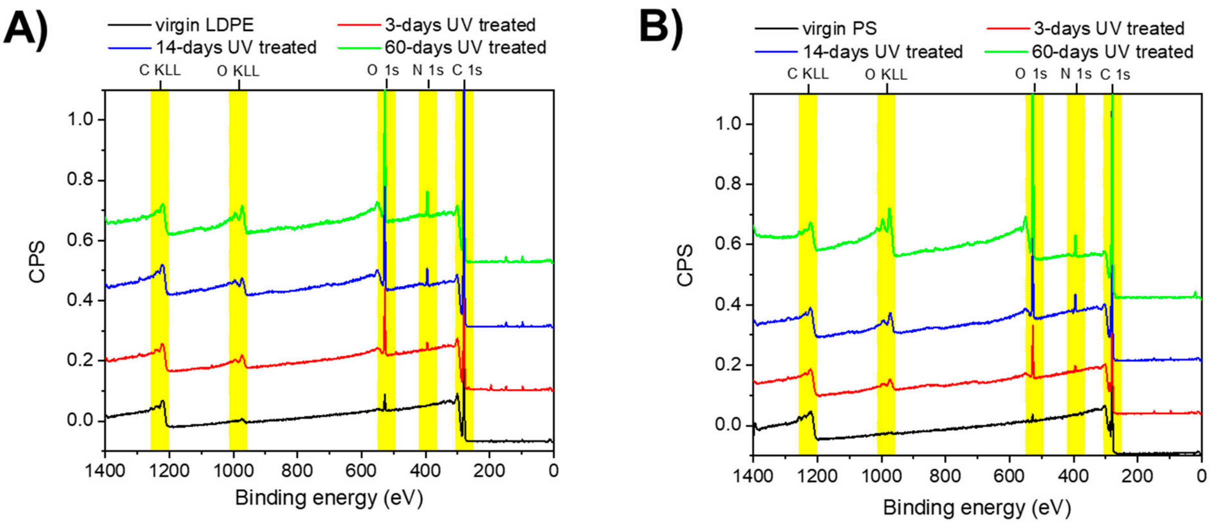


**Figure S1:** XPS wide spectra of LDPE (A) and PS (B) samples before and after 3, 14 and 60 days of UV-radiation artificial weathering.

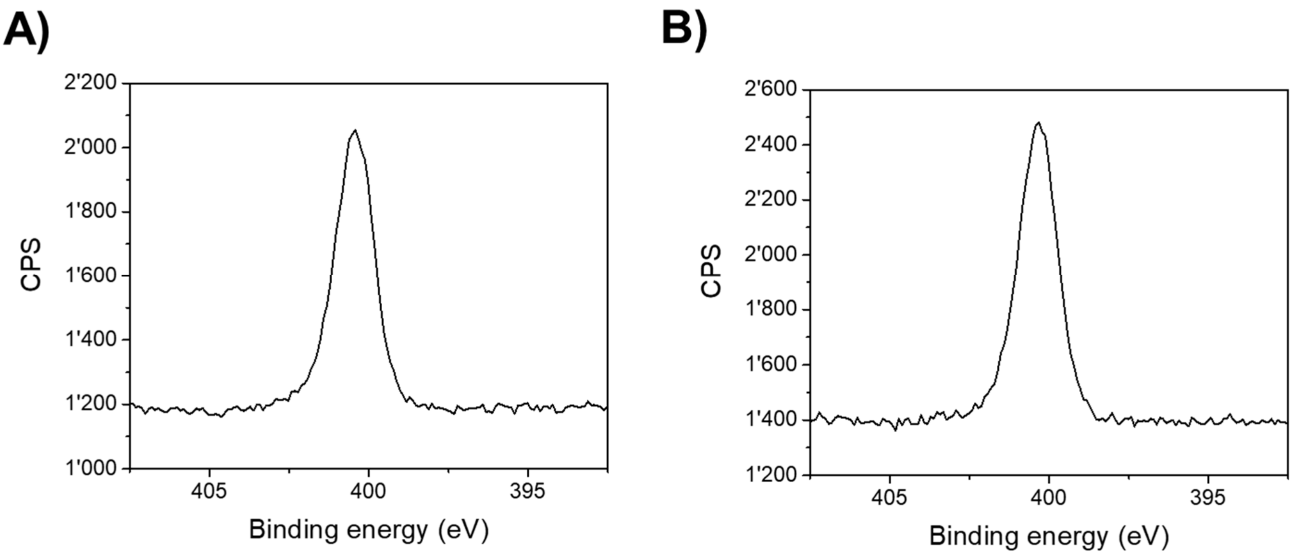


**Figure S2:** High-resolution N1s spectrum on LDPE (A) and PS (B) samples artificially weathered for 14 days.

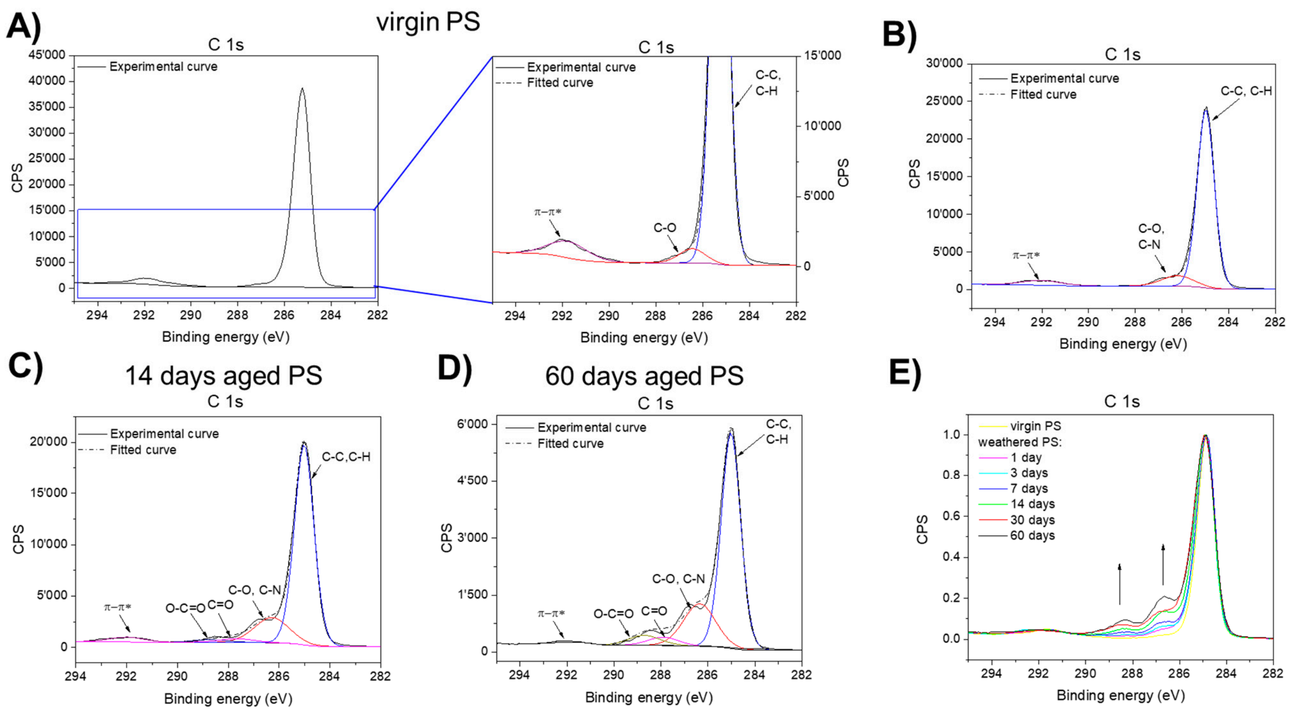


**Figure S3:** High-resolution spectra of C1s signal recorded for: virgin PS (A), 3-, 14- and 60-days aged PS (B, C and D, respectively). F) Comparison of C1s signals recorded on PS at different time weathering.

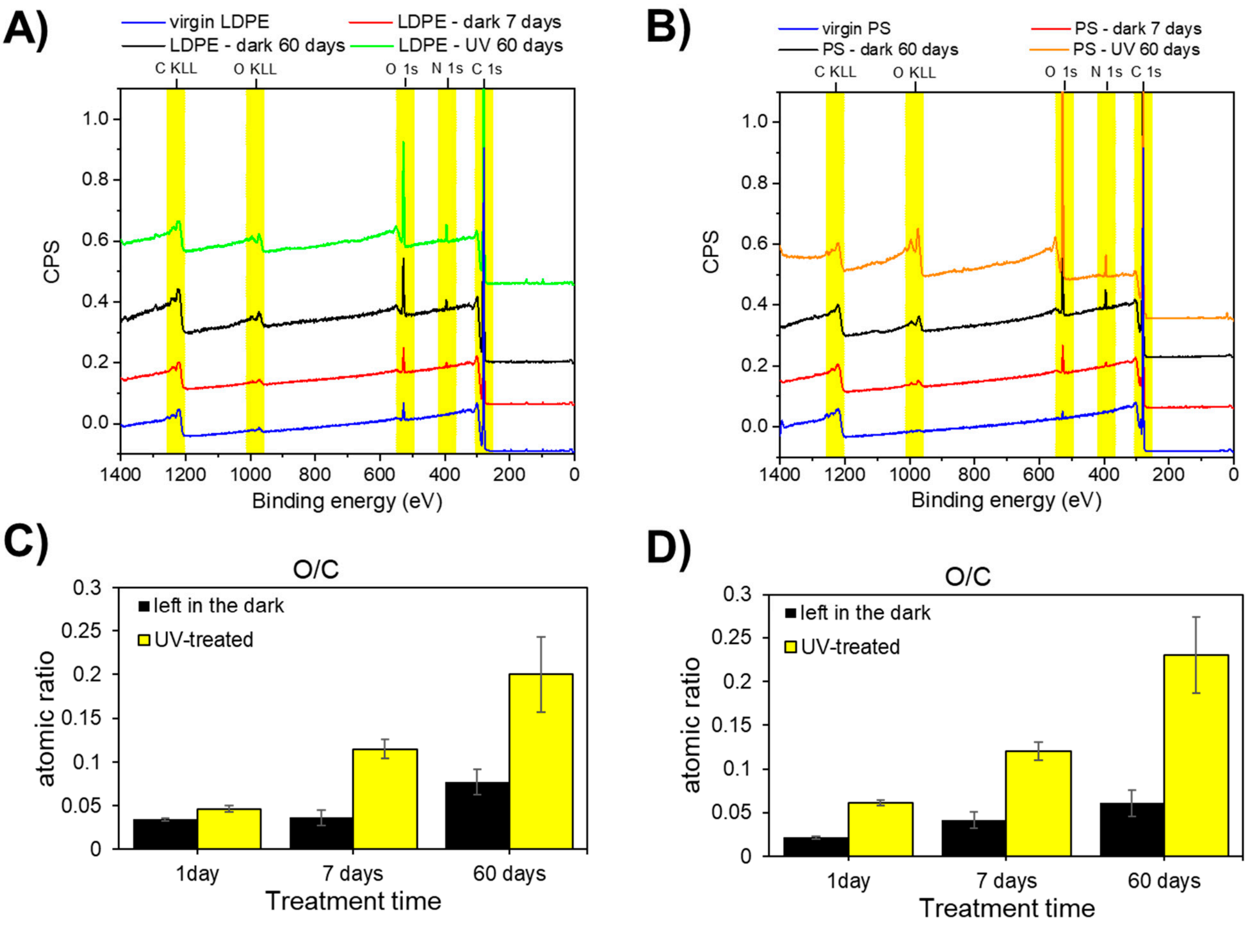


**Figure S4:** XPS wide spectra of LDPE (A) and PS (B) samples before and after 7, 60 days of artificial weathering at dark and 60 days under UV-radiation. O/C atomic ratio calculated from XPS analysis of LDPE (C) and PS (D) samples exposed to artificial weathering under UV-radiation (yellow bars) and at dark (black bars) for different time intervals.

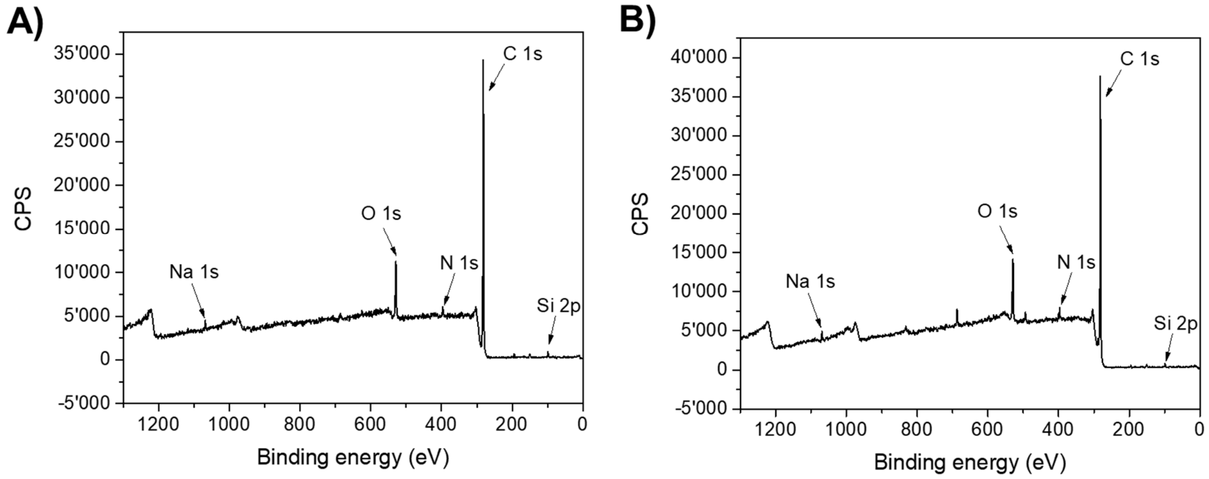


**Figure S5:** XPS wide spectra of LDPE (A) and PS (B) pellets recorded after their UV-weathering treatment (7 days) in marine water.

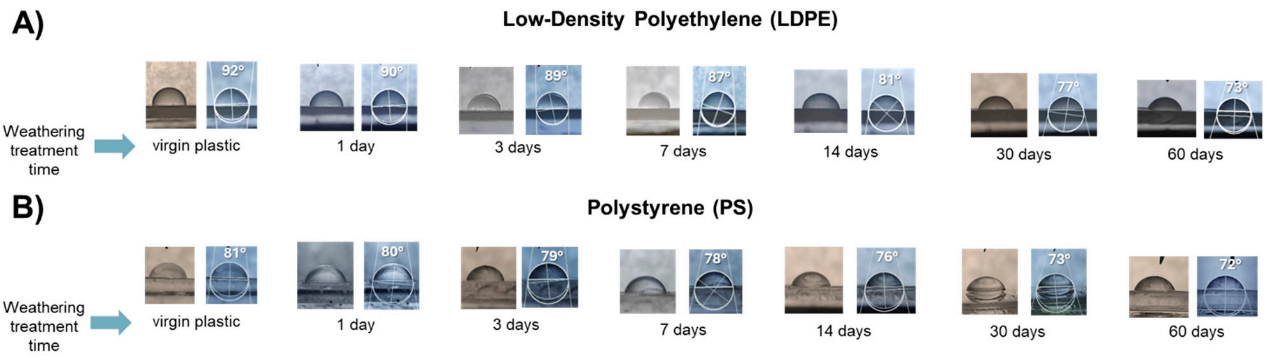


C)

| TREATMENT TIME (days) | CONTACT ANGLE - LDPE (°) | CONTACT ANGLE - PS (°) |
|---|---|---|
| 0 | 92.1 ± 0.9 | 81.4 ± 1.3 |
| 1 | 89.9 ± 0.5 | 80.5 ± 0.3 |
| 3 | 89.0 ± 2.1 | 79.8 ± 0.9 |
| 7 | 87.5 ± 1.4 | 78.3 ± 1.8 |
| 14 | 80.4 ± 0.6 | 76.3 ± 5.3 |
| 30 | 78.3 ± 2.3 | 73.5 ± 2.7 |
| 60 | 71.9 ± 5.3 | 73.2 ± 2.7 |

**Figure S6:** Results of contact angle measurement on LDPE (A) and PS (B) samples exposed to artificial weathering UV-treatment for different time intervals. C) Summary and comparison of the measured values.

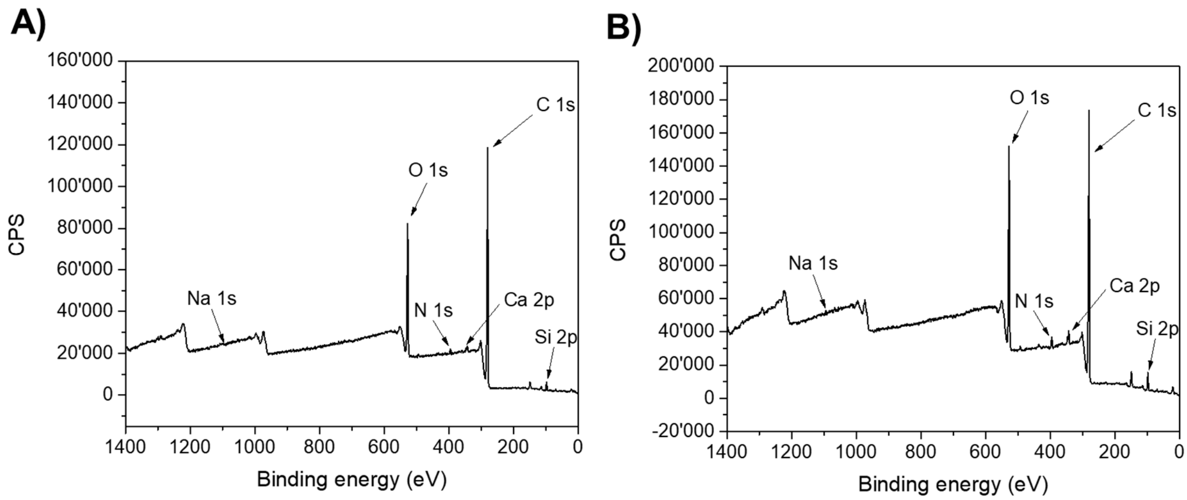


**Figure S7:** XPS wide spectra of LDPE foil (A) and PS cup (B) collected from “La Strea” beach (Italy).
